# Chorus Organization in a Neotropical Forest Cicada

**DOI:** 10.3390/biology13110913

**Published:** 2024-11-08

**Authors:** Guy Beauchamp

**Affiliations:** Independent Researcher, Montréal, QC, Canada; guygillesbeauchamp@gmail.com

**Keywords:** acoustic communication, coordination, insects, predation risk, synchronization

## Abstract

In many species of animals, males aggregate in particular locations and produce calls to attract searching females for reproduction. One striking feature of such choruses is synchronization. On a scale of hours, choruses are often concentrated at particular times of day, such as dawn or dusk. On a scale of seconds, males may also synchronize the rhythm of their calls with one another. While synchronized calling at this scale suggests benefits acting at the collective level, competitive interactions between males to attract females can also lead to synchronized calling as an epiphenomenon. Why males in some species synchronize the rhythm of their calls is still debated, and more work is needed to understand the evolution of this behavior. I investigated chorus organization in the Emerald cicada (*Zammara smaragdula*), a Neotropical forest cicada in southern Belize, to explore these issues. Choruses in this species occurred at dawn and dusk and, occasionally, during daytime. There was no evidence for synchronization in the rhythm of calls among males, as bouts of collective calling occurred after quiet intervals of variable rather than fixed durations. The temporal aggregation of calls in this species thus probably emerged from competitive interactions among males to attract females. The degree of temporal overlap in the calls of males during a chorus varied as a function of chorus phase and time of day, suggesting flexibility in chorus organization, perhaps in relation to temporal variations in factors such as the number of calling cicadas, the number of predators present or ambient temperature during a chorus.

## Simple Summary

Males in many species of animals congregate to produce calls that attract females for reproduction. Such choruses are often limited to particular times of day, and, in some cases, the rhythm of calls is synchronized among males, suggesting advantages at the collective level to calling at the same time. However, synchronized calling may also represent the outcome of competitive interactions among males without the need to invoke advantages at the collective level. I investigated chorus organization in one species of Neotropical forest cicada in southern Belize to explore these issues. Choruses of calling males occurred at dawn and dusk and occasionally during the daytime. I found no evidence of synchronization in the rhythm of calls by males, as bouts of calling occurred after variable rather than fixed quiet intervals. In this species, synchronized calling by males thus probably emerged from competitive interactions among males to attract females. The degree of temporal overlap in the calls of males during a chorus varied as a function of chorus phase and time of day, suggesting flexibility in chorus organization, perhaps in relation to variation in the environmental factors, such as the number of predators present or ambient temperature during a chorus.

## 1. Introduction

Males in many species of animals produce signals across a variety of sensory channels to attract females for reproduction [1]. Examples include auditory calls in insects and frogs [2] or visual flashing lights in fireflies [3]. Signals can be produced by solitary individuals, but males can also aggregate in particular locations to produce concurrent signals that attract searching females. This is the case in birds and mammals that aggregate in leks, which may include dozens of simultaneously displaying males [4], or in insects like cicadas, calling in large numbers from the same trees [5,6]. A striking feature of signals produced in such aggregations is synchronization [7], which can occur at different temporal scales. On a scale of hours, males can concentrate the production of their signals at particular times of the day. Choruses of calling males in insects, for instance, often take place at dawn or dusk [8]. Particular times of day may be preferred, because they offer the best conditions to send signals. Dawn and dusk, in particular, are quieter periods, and low ambient light might allow callers to avoid attracting unintended listeners like diurnal predators.

Synchronization in these aggregations may also occur at smaller time scales. At the scale of seconds, males may synchronize the rhythm of their signals with one another [7]. This happens in fireflies, where males in a given area gradually coordinate the timing of their flashing displays over time [9], and in insect choruses, where all males produce their calls at the same time [10]. Synchronized calls or visual displays at the aggregation level may act like beacons to attract searching females more effectively, may facilitate the identification of the signalling species by searching females or act to deter predation for the callers [7]. It is clear, however, that not all cases of synchronization at this scale have benefits acting at the collective level. Some cases of synchronization are actually considered epiphenomena of competitive interactions among males shaped by female preferences and may not necessarily have any adaptive value at the collective level [11]. In claw waving fiddler crabs (*Uca mjoebergi*), for instance, females prefer males in the aggregation that wave their claws first, leading to competitive interactions among males to signal first. Consequently, claw waving among males tends to cluster in time, with variable time intervals in between bouts of claw waving [12]. In general terms, temporally clumped signals may not necessarily imply that the rhythm of signal production is synchronized at the collective level.

Synchronization of rhythm in the production of calls is known in only a handful of species. In insects, synchronization of this type occurs in some Orthopterans like katydids and also in one species of periodical cicada [7]. Why some species synchronize the rhythm of their calls is still debated. Physiological constraints on call production, as well as ecological factors such as predation pressure, probably play a role in shaping call rhythm synchronization. However, our understanding is limited, and more work is needed with other species. This is especially relevant in overlooked geographical areas that might provide unique challenges to calling insects. To explore these issues, I investigated the occurrence of rhythm synchronization in the calls of a Neotropical insect species, the Emerald cicada (*Zammara smaragdula*). Many cicada species are found in the Neotropics [13], but their life history remains poorly studied [14]. Dawn and dusk choruses are known in some Neotropical cicadas [5,8]. However, the organization of their choruses at smaller time scales remains to be investigated in more detail.

If the rhythm of calls is synchronized in Emerald cicada choruses, I predicted that bouts of calling among individuals should be aligned temporally, and the duration of quiet intervals between calling bouts should show little variation. In addition, synchronization may only emerge after a tuning period, like in fireflies, and may be affected by environmental factors such as temperature and light levels, as is known in other species [6,15]. Therefore, I also examined call rhythm synchronization during different phases of the chorus and at different times of the day. If the synchronization of calls among males is less pronounced at particular times, temporal overlap in the calls of different males should be less extensive, so that the call of any one male may fall anywhere during the calls of others. In such a case, bouts of calling measured at the collective level will last longer, which provides us with a means to determine whether the extent of synchronization among calling males varies between different phases of choruses or among choruses produced at different times of the day.

## 2. Materials and Methods

### 2.1. Study Site and Subjects

I recorded Emerald cicada choruses in early April 2024 at the Belize Foundation for Research and Environmental Education research station located in Southern Belize (16.5° N, 88.7° W). The area consisted of a mosaic of early and late successional tropical rainforest near sea level. Local naturalists identified the chorusing cicada at the station as the Emerald cicada, which has a distinctive song heard clearly in all the recorded choruses. Cicada choruses were recorded at various locations located within 500 m of the station centre on different trails in an effort to sample different sets of individuals.

### 2.2. Sampling Procedure and Acoustic Measurements

I recorded choruses using the Merlin Bird ID app (https://merlin.allaboutbirds.org/download/, accessed on 1 April 2024), which is powered by smartphone recordings at a sampling frequency of 44.1 kHz. The recordings were in mono and were produced in .WAV format. I aimed the recording device directly at the canopy above the trails. As cicadas sang from tree trunks and/or the canopy, often at low light levels, the distance between the recording device and the chorusing cicadas could not be precisely determined. The temperature at the time of recordings varied between 21 °C and 34 °C, depending on the time of day. Dawn and dusk choruses occurred daily during the study period. I aimed to start recording these choruses as the first males started to call. I terminated recordings when the sound produced by choruses became faint 20–30 min later. Choruses also occurred during the daytime but not every day. I recorded daytime choruses opportunistically.

**Figure 1:**
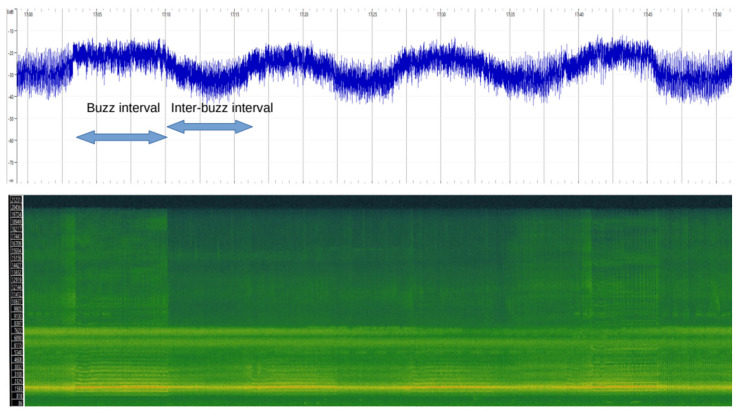
Sample of calls in a chorus of the Emerald cicada. The top panel illustrates amplitude on the dB scale as a function of time. The bottom panel shows the spectrogram in the frequency domain, with the *y*-axis corresponding to frequency (Hz). The power of each frequency within the timeframe is shown by the brightness of the pixels corresponding to that frequency. At the collective level, the recordings show buzz intervals with a high amplitude and inter-buzz intervals in between, where the characteristics frequencies associated with buzzing are not present. The duration of one buzz interval and of one inter-buzz interval is shown with arrows.

Acoustic measurements were made on each recording lasting at least 10 min. Measurements in the temporal and frequency domains were taken with Sonic Visualizer V. 5.0 (https://www.sonicvisualiser.org/, accessed on 1 April 2024). Calls consisted of a rapidly increasing train of pulses (sounding like ticks) followed by a lengthy trill or stridulation. This stridulation was the longest part in the call and is referred to here as the buzz interval. As the recordings included many different individuals, it was not clear when each individual started or terminated their calls. However, the buzz intervals at the collective level were clearly visible in the sonograms, both in terms of amplitude and frequencies (Figure 1).

Therefore, I measured the duration of each buzz interval from the moment the distinct frequencies associated with this part of the call became apparent to the moment these frequencies ended. The interval between two successive buzz intervals was also measured and is called the inter-buzz interval. In addition to these two intervals, I also measured the amplitude during each buzz interval. In a buzz interval, the amplitude ranged over several decibels (Figure 1). Lining up the sonogram with the amplitude scale provided by the software, I noted the approximate decibel value associated with the mid-point on the amplitude range. Other animals were calling at the time of the recordings and thus added to the sound amplitude. However, their contribution to the overall sound amplitude was considered negligible, given the overwhelming loudness of the cicada choruses.

**Figure 2:**
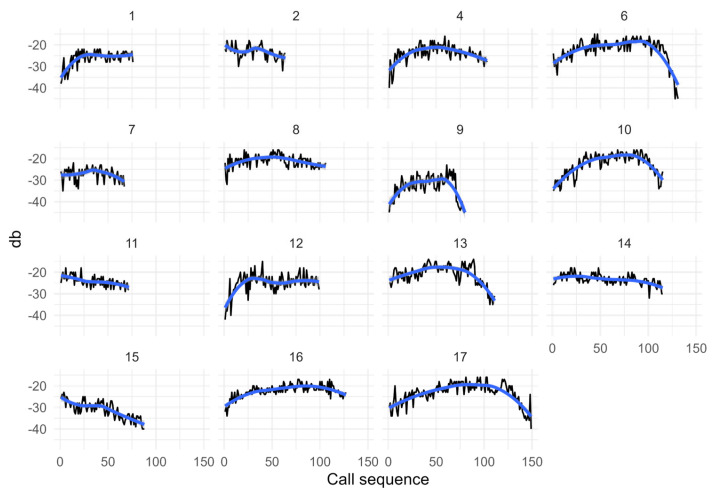
Sound amplitude variation (dB) for all recordings of choruses in the Emerald cicada. Labels at the top of each panel refer to chorus ID. Sound amplitude was measured for each buzz interval during the duration of a chorus. Higher values on the *y*-axis indicate louder choruses. The *x*-axis shows the sequence of buzz intervals during a chorus. The trend line in blue shows the smoothed conditional mean.

Typically, the sound amplitude during a chorus increased in time, remained high for several minutes, and eventually decreased to reach low levels. I refer to these different parts of the chorus as the increasing, plateau and decreasing phases (Figure 2). I used inflection points in plots of amplitude versus time for each recording to determine approximately the beginning and end of each phase. Notice that, in a few recordings, I missed the increasing or decreasing phase altogether or parts of the plateau phase. I was not able to ascertain the age of the calling cicadas. As the call features might change with age, I made the assumption that age composition in the different recorded choruses was similar across chorus phases and at different times of day.

### 2.3. Statistical Analysis

The unit of analysis was the recording of a chorus made at a particular time and a particular location. It was not possible to isolate individual calls. The output of each recording was thus the sum of all calls recorded within the range of the smartphone. Each recording was given a unique ID. During the plateau phase, where call measurements are expected to be relatively stable across time, I calculated the mean, standard deviation and coefficient of variation (CV = 100 × SD/Mean) of the buzz and inter-buzz interval durations. As a measure of variability, I obtained the intra-class correlation coefficient (ICC) for buzz interval durations, inter-buzz interval durations and call duration at the collective level (the sum of the above two intervals) using variance components from a linear mixed model, including time of day as a fixed factor and chorus ID as a random effect. ICC values can range between 0 and 1, with values closer to 0 indicating that measurements are less repeatable.

For inferential statistics, I first examined the effect of the time of day (early morning = dawn, daytime outside of dawn or dusk and late evening = dusk) on sound amplitude, as defined above, for each buzz interval during the plateau phase of each recording using a linear mixed model, with chorus ID as a random effect and time of day as a fixed effect. Second, I examined the effect of the time of day and chorus phase (increasing, plateau and decreasing) on buzz interval durations and on inter-buzz interval durations using a linear mixed model with chorus ID as a random effect and time of day, chorus phase and the interaction between time of day and chorus phase as fixed effects. Due to skewness in the distribution of inter-buzz intervals and call duration, I used log_10_ transformation to normalize the residual values for these variables.

## 3. Results

I obtained 15 recordings of Emerald cicada choruses ranging in duration from 13 to 34 min, including five choruses at each time of day. From these recordings, I timed the duration of 1502 buzz intervals and 1489 inter-buzz intervals. In longer recordings, different phases were apparent during a chorus. The increasing phase lasted typically less than 10 min, with the sound amplitude rising sharply over time (Figure 2). The decreasing phase lasted typically less than 5 min, and the sound amplitude near the end reverted back to the low levels recorded at the beginning of the chorus.

**Figure 3:**
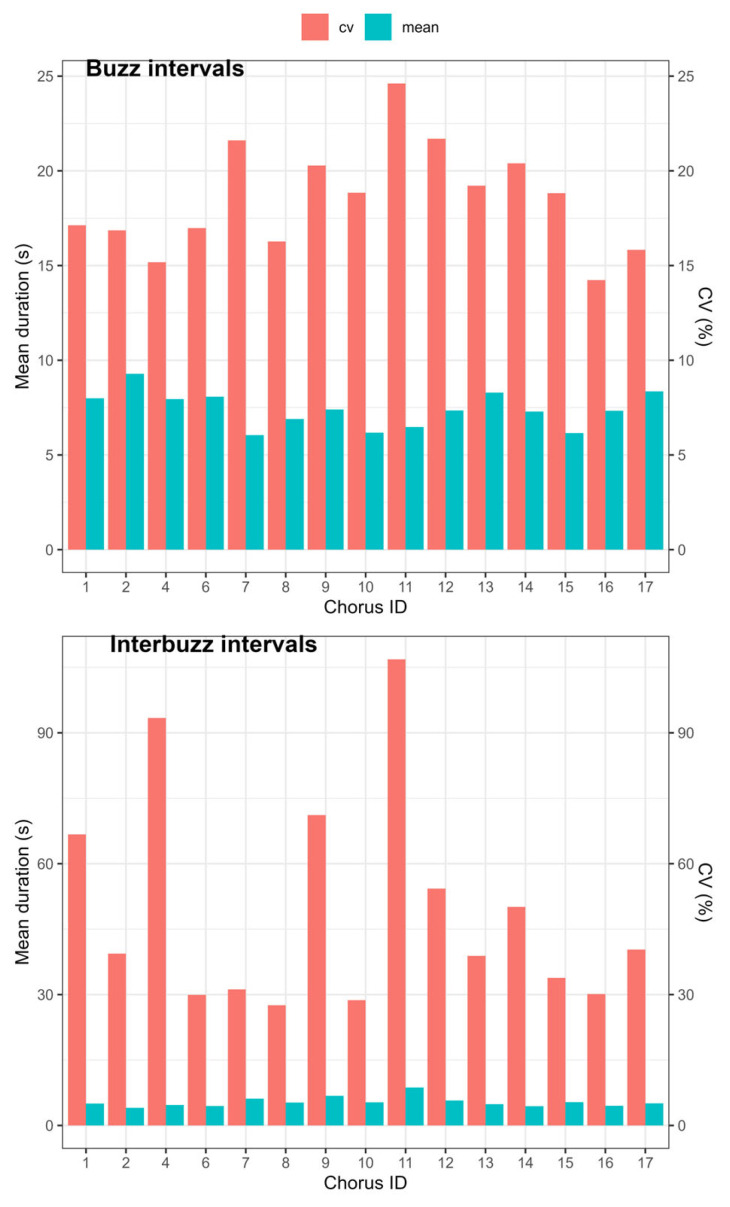
Mean and coefficient of variation (%) of the duration of the buzz and inter-buzz intervals in different choruses of the Emerald cicada.

During the plateau phase, a typical buzz interval lasted approximately 8 s and a new buzz interval typically followed after 5 s (Figure 3). The plateau phase consisted of a succession of such buzz intervals followed by quieter periods of various durations. Indeed, the variation in duration among inter-buzz intervals in a chorus, as measured by CV, was two to three times larger than in buzz intervals (Figure 3). The ICC for call duration on the log_10_ scale was 0.036 (95% CI: 0.0021, 0.082), indicating low repeatability within a chorus for this measurement. Repeatability was low for inter-buzz interval durations at the log_10_ scale (R = 0.059, 95% CI: 0.011, 0.12) and slightly higher for buzz interval durations (R = 0.12, 95% CI: 0.039, 0.22). The mean sound amplitude did not vary significantly as a function of the time of day (F_2,11.9_ = 0.62, *p* = 0.55) during the plateau phase.

**Figure 4:**
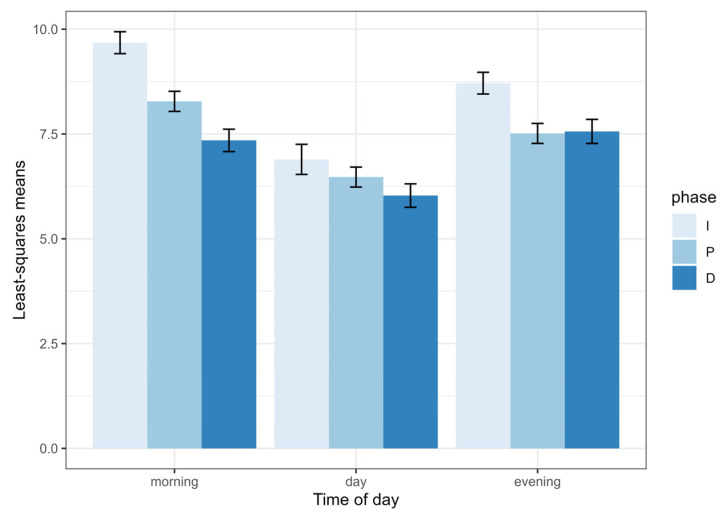
Least squares means of buzz interval durations (s) in choruses of the Emerald cicada as a function of time of day and chorus phase (I = increasing, P = plateau and D = decreasing). Bars show one standard error.

Using all three phases of the choruses, the linear mixed model revealed a significant effect on the time of day (F_2,12.9_ = 17.5, *p* = 0.0002), on the chorus phase (F_2,1473.6_ = 50.9, *p* < 0.0001) and on the interaction between the time of day and chorus phase (F_4,1465.3_ = 6.4, *p* < 0.0001) on the duration of buzz intervals (Figure 4).

I performed post hoc tests with the sequential Benjamini–Hochberg adjustment procedure to identify differences between different levels of these variables. During early morning choruses, the duration of buzz intervals in the plateau phase was significantly shorter than during the increasing phase but longer than during the decreasing phase. During daytime choruses, the duration of buzz intervals did not vary as a function of the chorus phase. During evening choruses, the duration of buzz intervals was longer during the increasing phase than during the two other phases. In terms of the time of day, the duration of buzz intervals was shorter during the daytime than during the early morning and the late evening at all three phases of the chorus, except for the plateau phase, where there was no difference between daytime and late evening. There were no differences between early morning and late evening choruses at any of the three phases.

**Figure 5:**
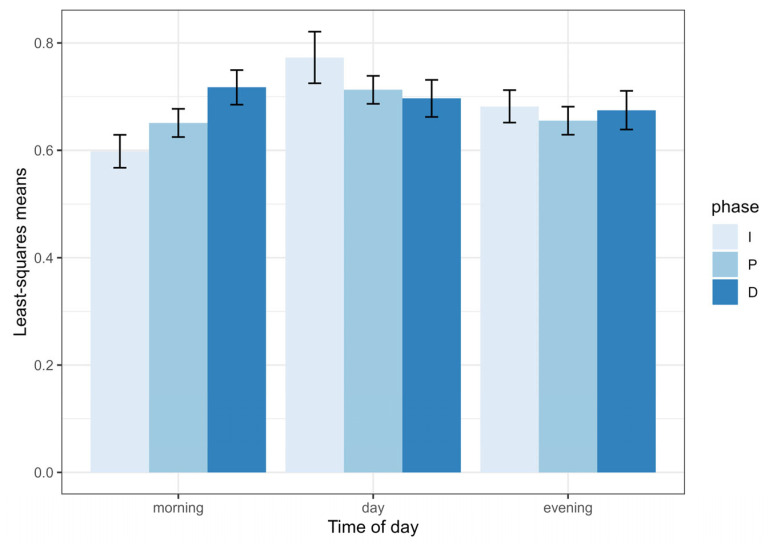
Least squares means in a log_10_ scale of inter-buzz interval durations (s) in choruses of the Emerald cicada as a function of the time of day and chorus phase (I = increasing, P = plateau, D = decreasing). Bars show one standard error.

Using all three phases of the choruses, the linear mixed model revealed no significant effect of the time of day (F_2,13.5_ = 17.5, *p* = 0.18) or chorus phase (F_2,1349.1_ = 1.0, *p* = 0.37) on the duration of inter-buzz intervals. There was a significant interaction between time of day and chorus phase (F_4,1332.4_ = 3.9, *p* = 0.004) (Figure 5). Post hoc tests revealed only one significant difference: the duration of inter-buzz intervals was significantly longer during the descending than during the increasing phase in early morning choruses.

## 4. Discussion

As in other Neotropical cicadas, the Emerald cicada in Southern Belize produced choruses at dawn and dusk and also occasionally during the daytime [5,8,16]. Typically lasting about half an hour, these choruses evolved from faint to nearly deafening, as more and more local cicadas joined in. Lacking the means to localize individual cicadas at low light levels in the dense vegetation, it was not clear how many cicadas joined the choruses. Choruses could be heard anywhere in a radius of at least 500 m at the field station at dawn and dusk but appeared more localized during the daytime. The sound amplitude of the choruses during the plateau phase did not vary significantly as a function of the time of day, suggesting that the various recordings involved similar numbers of cicadas.

This study revealed that the rhythm of calls in Emerald cicada choruses was not synchronized among individuals on a scale of seconds. During the plateau phase of the choruses, where the maximum sound amplitude was documented, bouts of calling occurred at irregular intervals ranging from a few seconds to more than 20 s. With the call rhythm synchronization, the interval between bouts of calling should be regular in duration and therefore highly repeatable within choruses. Indeed, in species with rhythm synchronization, individuals maintain a tight control over the tempo of calling [7], suggesting that repeatability of call measurements within choruses should be closer to 1 rather than 0. However, repeatability within choruses was low for inter-buzz interval durations, as well as for call durations, suggesting that most of the variations in these measurements occurred within, rather than between, choruses, controlling for the time of day. These results fit the pattern observed in some frog choruses, where quiet intervals between calling bouts are the most variable part of choruses [17]. Asynchronous choruses are also known in other insects [16]. In cicadas, only one species is known to synchronize the rhythm of their calls [6], but little work has focused on chorus organization in cicadas as opposed to other taxa like Orthopterans and Anurans.

The degree of overlap in calls during a chorus, as a measure of synchronization among individuals, varied as a function of chorus phase and time of day. The longest buzz intervals at the collective level occurred in the early phases of a chorus where low sound amplitude suggests that few individuals were calling at that time. Given that this pattern was observed at dawn and dusk, the results suggest that it was not low light that prevented individuals from joining the emerging chorus. Lack of organization implies that calls of different individuals were loosely overlapping temporally yielding longer calling bouts at the collective level. This finding is similar to the observation that the synchronization of flashing lights in fireflies only emerges gradually [9]. One possibility that cannot be addressed here is that males called at greater distances from one another in the increasing phase of a chorus and perhaps paid less attention to more distant males, yielding less temporal overlap in calls. An effect of distance between males on calling behavior was noted in other species [17,18]. During the decreasing phase of a chorus, the buzz intervals were shorter than during the plateau phase. Given the low sound amplitude at that time, my impression was that few individuals were still calling so the overlap between calls was very slight, yielding short buzz intervals.

Buzz intervals at the collective level were the shortest during daytime choruses, with no difference between dawn and dusk, suggesting more overlap between calls of different males at that time of day. Given that the sound amplitude of choruses recorded at different times of the day did not vary, differential recruitment to choruses as a function of time of the day was not a confounding effect. Environmental effects on the synchronization of calls or displays in general are known in other species. When more light reaches the canopy, for instance, periodical cicadas are more likely to synchronize their calls [6]. By contrast, synchronization decreased for sea fireflies when more light penetrated the water [15]. Why environmental factors such as light influence the chorus features is not clear. In frogs, more synchronous calling is thought to deter predation by bats [19]. It is possible that more temporal overlap in the calls of cicadas during the daytime is a response to diurnal predators more likely to be active at this time of day than at dawn or dusk, where light levels are low. As little is known about possible predators of cicadas in Belize and elsewhere in general, this idea remains conjecture. Another possibility is that higher temperatures during the daytime favored shorter calls. Buzzing, which involves the contraction of muscles in the abdomen, produces excess heat that might cause a thermoregulation problem at higher ambient temperatures [20]. Nevertheless, cicadas in general possess various means of controlling their body temperature [21], casting doubt on this hypothesis. Future studies might focus on choruses occurring during other seasons to determine whether choruses produced at different ambient temperatures are characterized by different degrees of call overlap.

In terms of limits, this study was conducted over a short period of time, and thus, it is not clear whether the patterns in chorus organization documented here also apply to other seasons with potentially different environmental conditions. The inability to identify individual calls makes it difficult to determine the strategies used by different individuals to compete with other males in choruses. Other studies have shown, for instance, that males adopt strategies to call the first more often [11], as females show a preference for leading calls in a collective bout of calling.

## 5. Conclusions

Choruses of Emerald cicadas in Southern Belize were synchronized on a scale of hours, as they tended to occur predominantly at dawn and dusk. However, the rhythm of calling among males in these choruses was not synchronized on a scale of seconds, fitting the idea that temporal aggregation of calls can emerge from competitive interactions among males to attract females. The degree of call synchrony among males during a chorus varied as a function of the chorus phase and time of day, suggesting flexibility in chorus organization, perhaps in relation to temporal variations in the environmental factors, such as the number of calling cicadas, the number of predators present or ambient temperature during a chorus.

## Data Availability

The data are available from the corresponding author.

## References

[1] Bradbury J.W., Vehrencamp S.L. (2011). Principles of Animal Communication.

[2] Gerhardt H.C., Huber F. (2002). Acoustic Communication in Insects and Anurans: Common Problems and Diverse Solutions.

[3] Carlson A.D., Copeland J. (1985). Flash communication in Fireflies. Q. Rev. Biol..

[4] Hoglund J., Alatalo R.V. (1995). Leks.

[5] Sueur J. (2008). Cicada acoustic communication: Potential sound partitioning in a multispecies community from Mexico (Hemiptera: Cicadomorpha: Cicadidae). Biol. J. Linn. Soc..

[6] Sheppard L.W., Mechtley B., Walter J.A., Reuman D.C. (2020). Self-organizing cicada choruses respond to the local sound and light environment. Ecol. Evol..

[7] Greenfield M.D., Merker B. (2023). Coordinated rhythms in animal species, including humans: Entrainment from bushcricket chorusing to the philharmonic orchestra. Neurosci. Biobehav. R..

[8] Young A.M. (1981). Temporal selection for communicatory optimization: The dawn-dusk chorus as an adaptation in tropical cicadas. Am. Nat..

[9] Buck J. (1988). Synchronous rhythmic flashing of fireflies. II. Q. Rev. Biol..

[10] Greenfield M.D. (2015). Synchronous and alternating choruses in insects and anurans: Common mechanisms and diverse functions. Am. Zool..

[11] Greenfield M.D., Roizen I. (1993). Katydid synchronous chorusing is an evolutionarily stable outcome of female choice. Nature.

[12] Reaney L.T., Sims R.A., Sims S.W., Jennions M.D., Backwell P.R. (2008). Experiments with robots explain synchronized courtship in fiddler crabs. Curr. Biol..

[13] Sanborn A.F. (2006). New records for the cicada fauna from four Central American countries (Hemiptera: Cicadoidea: Cicadidae). Florida Entomol..

[14] Acosta R.C., Ruschel T.P., Kaminski L.A. (2024). Flying singers: Spatio-temporal distribution and acoustic dynamics of two species of Carinetini (Hemiptera: Cicadidae) cicadas in sympatry. Zool. J. Linn. Soc..

[15] Hensley N.M., Rivers T.J., Gerrish G.A., Saha R., Oakley T.H. (2023). Collective synchrony of mating signals modulated by ecological cues and social signals in bioluminescent sea fireflies. Proc. R. Soc. Lond B Biol. Sci..

[16] Greenfield M.D. (1983). Unsynchronized chorusing in the coneheaded katydid *Neoconocephalus affinis* (Beauvois). Anim. Behav..

[17] Larter L.C., Ryan M.J. (2024). Túngara frog call-timing decisions arise as internal rhythms interact with fluctuating chorus noise. Behav. Ecol..

[18] Cocroft R.B., Pogue M. (1996). Social behavior and communication in the Neotropical Cicada *Fidicina mannifera* (Fabricius) (Homoptera: Cicadidae). J. Kansas Entomol. Soc..

[19] Tuttle M.D., Ryan M.J. (1982). The role of synchronized calling, ambient light, and ambient noise, in anti-bat-predator behavior of a treefrog. Behav. Ecol. Sociobiol..

[20] Sanborn A.F. (2001). Timbal muscle physiology in the endothermic cicada *Tibicen winnemanna* (Hemiptera: Cicadidae). Comp. Biochem. Physiol. Part A Mol. Integr. Physiol..

[21] Sanborn A.F. (2004). Thermoregulation and endothermy in the large western cicada *Tibicen cultriformis* (Hemiptera: Cicadidae). J. Therm. Biol..

